# Debunking the Concept of Dentinal Tubule Penetration of Endodontic Sealers: Sealer Staining with Rhodamine B Fluorescent Dye Is an Inadequate Method

**DOI:** 10.3390/ma14123211

**Published:** 2021-06-10

**Authors:** David Donnermeyer, Sina Schmidt, Arno Rohrbach, Johannes Berlandi, Sebastian Bürklein, Edgar Schäfer

**Affiliations:** 1 Department of Periodontology and Operative Dentistry, Westphalian Wilhelms-University, Albert-Schweitzer-Campus 1, Building W 30, 48149 Münster, Germany; sina.schmidt@ukmuenster.de; 2 Institute for Mineralogy, Westphalian Wilhelms-University, Correnstraße 24, 48149 Münster, Germany; rohrbaa@uni-muenster.de; 3 Institute of Neuropathology, University Hospital Münster, 48149 Münster, Germany; johannes.berlandi@ukmuenster.de; 4 Central Interdisciplinary Ambulance in the School of Dentistry, Albert-Schweitzer-Campus 1, Building W 30, 48149 Münster, Germany; sebastian.buerklein@ukmuenster.de (S.B.); eschaef@uni-muenster.de (E.S.)

**Keywords:** AH Plus, BioRoot RCS, confocal laser scanning microscope, scanning electron microscope, sealer penetration, Total Fill BC Sealer

## Abstract

The aim of this study was to investigate the suitability of rhodamine B dye staining of an epoxy resin sealer (AH Plus) and calcium-silicate-based sealers (Total Fill BC Sealer, BioRoot RCS) to represent the penetration depth of the sealers into dentinal tubules after root canal obturation. In a three-step process, (1) leaching of rhodamine B from sealers into a buffer solution, (2) passive penetration of leached rhodamine B into dentinal tubules, and (3) conformity of rhodamine B penetration assessed by confocal laser scanning microscopy (CLSM), and sealer penetration assessed by scanning electron microscopy (SEM), in root-canal-filled teeth, were evaluated. Rhodamine B dye massively leached out of Total Fill BC Sealer and BioRoot RCS into the phosphate-buffered saline (PBS). A pinkish coloration of AH Plus was found after contact with PBS. Leached rhodamine B dye passively penetrated dentinal tubules from all three sealers when placed on root dentin. No correlation was observed between sealer penetration in SEM and rhodamine B penetration in CLSM. Staining of sealers using rhodamine B is an inadequate method with which to evaluate sealer penetration depth into dentinal tubules, as it overestimates the penetration of sealers into root dentin tubules.

## 1. Introduction

Endodontic sealers should fill irregularities alongside the root canal wall and penetrate the dentinal tubules to ensure, together with a solid core material, a long-lasting, bacteria-tight seal of the root canal system [1]. Any remaining bacteria are entombed and become inactive, and the root canal system is preserved against reinfection [2]. This simple statement is a central dogma of endodontology and practical endodontics. However, what about the penetration of root canal sealers into dentinal tubules in detail? Numerous studies have evaluated this topic [3,4,5,6]. Endodontic sealers of all kinds were labelled using organic fluorescent molecules such as rhodamine B or fluorescein [3,4]. Afterwards, impressive images of root canal slices show the fluorescent sealers’ penetration deep into the root dentin along the dentinal tubules. The impression of an inextricable interweaving of the root canal sealer and the root dentin is apparent, and it is often claimed that this interweaving is one key to the success of root canal treatment. Hence, why is there no detectable change in the radiopacity of the root dentin in the final control radiograph after root canal obturation, when a vast number of dentinal tubules must be filled with a radiopaque sealer at this point? The clinical occurrence of a lateral canal or apical ramification with a slight sealer extrusion is a rare observation [7]. The theoretical number of side canals is higher than the number of obturated side canals [8]. If the pressure on the sealer during root canal filling is not high enough to fill all auxiliary canals, how can the sealer be pressed into much smaller dentinal tubules?

To evaluate sealer penetration into dentinal tubules, confocal laser scanning microscopy (CLSM) with the use of fluorescent organic dyes has evolved to become the standard [3,4,6]. CLSM has advantages compared to other magnification methods because it does not evaluate the surface of the specimen, but can display the situation of different levels underneath the specimen’s surface. Therefore, CSLM is not dependent on surface quality, and no surface preparation that could cause artifacts is necessary [9]. As a major disadvantage, CLSM cannot display non-fluorescent materials directly. In all cases, materials must be stained with fluorescent dyes that bind to the materials.

Considering a lack of correlation between clinical observations, such as increased root dentin radiopacity, and in vitro penetration of the sealer into dentinal tubules, doubts were raised about the widespread methodology. Already, in 2017, Jeong et al. speculated on the moisture affinity of rhodamine B and the possible leaching from the sealer into the dentinal tubules. An inaccuracy, showing deeper penetration of rhodamine B than the sealer, was feared [10]. Therefore, in their study, a fluorescent calcium tracer—Fluo-3 pentaamonium salt—was used to evaluate the penetration depth, using CLSM. The same method was also reported regarding the penetration depth of calcium-silicate-based sealers [11]. Lately, a discrepancy between the use of rhodamine B and Fluo-3 pentaamonium to precisely indicate the sealer’s penetration depth was reported for calcium-silicate-based sealers [12]. To date, no direct correlation between rhodamine staining and intratubular sealer has undergone investigation.

The present study was hence designed to investigate the suitability of fluorescent staining of epoxy-resin- or calcium-silicate-based sealers, in order to represent their penetration depth into dentinal tubules after root canal obturation. The experimental design followed a three-step process to gather information about the suitability of rhodamine B dye for effective and long-lasting staining of these sealers. The first experimental setup aimed to investigate whether rhodamine B dye would leach from a mixture with different sealer types. The second experimental setup aimed to evaluate whether leached rhodamine could penetrate passively into dentinal tubules. The aim of the third experimental setup was to compare SEM and CLSM images of root slices, in order to evaluate the conformity of these methods. The null hypothesis was that rhodamine B dye would effectively stain root canal sealers, and thereby is a reliable marker for dentinal tubule penetration of root canal sealers.

## 2. Material & Methods

### 2.1. Experimental Setup 1

The sealers AH Plus (Dentsply Sirona, York, PA, USA), Total Fill BC Sealer (FKG Dentaire, La Chaux-de-Fonds, Switzerland), and BioRoot RCS (Septodont, St. Maur des Fossés, France) were mixed on a glass plate with rhodamine B at a ratio of 0.1% by weight (*n* = 3 per sealer). Rhodamine B powder (Merck, Darmstadt, Germany) was used in all experiments. AH Plus and BioRoot RCS were mixed directly beforehand, as instructed by the manufacturers, on the glass plate. Total Fill BC Sealer was dispensed directly on a glass plate and mixed with rhodamine B. AH Plus was filled in stainless steel molds (height 1 mm, diameter 10 mm) as demanded in the ISO 6876 specifications, while the calcium-silicate-based sealers were filled in plaster molds of the same size. The plaster molds were kept in a water bath for 24 h beforehand, in order to ensure sufficient moisture supply to allow these sealers to set. The coloration of the specimens was documented by photography (T_0_). All of the molds were stored in an incubator (Memmert, Schwabingen, Germany) at 37 °C and 100% humidity for 24 h, in order to ensure setting of the sealers [13,14,15]. After 24 h, the molds were inserted into 50 mL beakers, and 30 mL phosphate-buffered saline (PBS) was added. The specimens were once again stored in the incubator under the same conditions for 7 days, and the coloration of the buffer solution and the sealer was documented by photography (T_1_). The photographs were compared blind in order to evaluate whether the sealers could be permanently stained with rhodamine B.

### 2.2. Experimental Setup 2

The passive penetration of rhodamine B dye into dentinal tubules was evaluated after the fluorescent dye was eventually washed out of the sealer (*n* = 3 per sealer). Root dentin sections 4 × 4 mm in size were obtained from cervical root dentin from extracted upper third molars (Enretec.dental, Velten, Germany), in accordance with the Declaration of Helsinki. The sections were prepared using a diamond cutting disc (Komet, Lemgo, Germany) and embedded into resin (Technovit 4071, Heraeus Kulzer, Hanau, Germany), and specimens with a height of 1 mm were prepared using a water-cooled diamond saw (Leitz, Wetzlar, Germany). The dentin specimens were cleaned in an ultrasonic bath in 3% NaOCl, 17% EDTA, and distilled water for 5 min each in order to ensure the complete removal of debris. The sealers were prepared with rhodamine B dye, as described in the previous step, and a portion of 0.15 g of sealer was placed on top of the dentin specimen. All specimens were set afloat on 30 mL PBS in a beaker and stored in an incubator at 37 °C and 100% humidity for 14 days. After incubation, the sealers were removed from the specimens with a scalpel, and the specimens were again embedded into resin (Technovit 4071, Heraeus Kulzer, Hanau, Germany). Vertical sections of the specimens were prepared using a water-cooled diamond saw with a thickness of 1 mm. The specimen sections were evaluated for the penetration depth of the rhodamine B dye into the dentinal tubules using CLSM (excitation wave length 555 nm, LSM 700, Zeiss, Oberkochen, Germany).

### 2.3. Experimental Setup 3

Root canals were obturated with the stained sealers in order to evaluate sealer and dye penetration in comparison with previously published studies. Nine central upper incisors (Enretec.dental, Velten, Germany) were selected (*n* = 3 per sealer), in accordance with the Declaration of Helsinki. The roots were checked under a light microscope (Expert DN, Müller Optronic, Erfurt, Germany) in order to exclude roots with fractures or resorption. Radiographs were taken from the mesiobuccal and buccolingual directions in order to ensure that all of the teeth had only one root canal. The incisors were coronally cut to a standard length of 20 mm. After access cavity preparation, the root canals were instrumented with ProTaper Gold Rotary Files (Dentsply Sirona, York, PA, USA) up to size F3 under irrigation with 3% NaOCl. Finally, all canals were rinsed with 5 mL NaOCl, 5 mL 17% EDTA, and 5 mL 0.9% NaCl. EDTA was activated for 30 s using an ultrasonic tip (Irri-S, VDW, München, Germany) in an ultrasonic device at the 30% setting (VDW.Ultra, VDW, München, Germany). After mixing the sealers with rhodamine B dye, as described above, and drying the root canals with sterile paper points (Dentsply Sirona), the sealers were inserted into the canals. The canals were obturated with gutta-percha cones matching the F3 file (Dentsply Sirona, Charlotte, NC, USA), using the single-cone technique [6,10]. As rhodamine B leached from all sealer types in setup 2, the implementation of a negative control group was impossible. The access cavities were adhesively sealed using OptiBond FL Dental Adhesive (Kerr, Rastatt, Germany) and Estellite Sigma Quick composite resin (Tokuyama, Tokyo, Japan). The roots were covered with moistened gauze and kept in closed plastic vials filled with PBS in an incubator at 37 °C for 14 days. Afterwards, the roots were embedded into resin (Technovit 4071), and horizontal slices with a thickness of 1 mm were prepared at 5–6 mm from the root apex. The slices were evaluated for dentinal tubule penetration of the rhodamine B dye using CLSM (excitation wave length 555 nm, LSM 700, Zeiss). After 3 months of exsiccation in a closed container, the specimens were investigated for sealer penetration into the dentinal tubules using a scanning electron microscope (SEM) (JSM 6510 LA, JEOL, Freising, Germany). All sealers could be detected via EDX (Energy-dispersive X-ray spectroscopy) analysis for zirconium oxide, which is a radiopaque ingredient in all three sealers. The furthest penetration depth was measured using the software tools (SEM Control User Interface (version 3.13), JEOL; ZEN2011 (version 7.0.7.288), Zeiss, Aalen, Germany) of the two microscopes, and the values were compared.

## 3. Results

### 3.1. Experimental Setup 1

**Figure 1:**
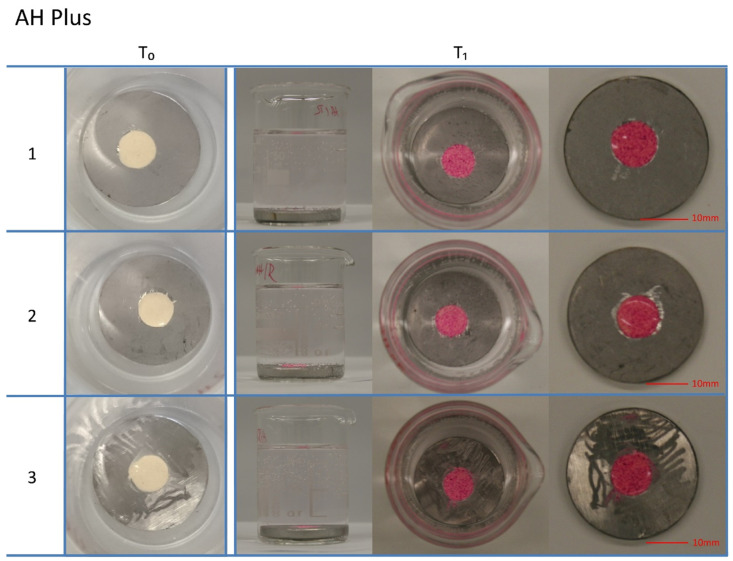

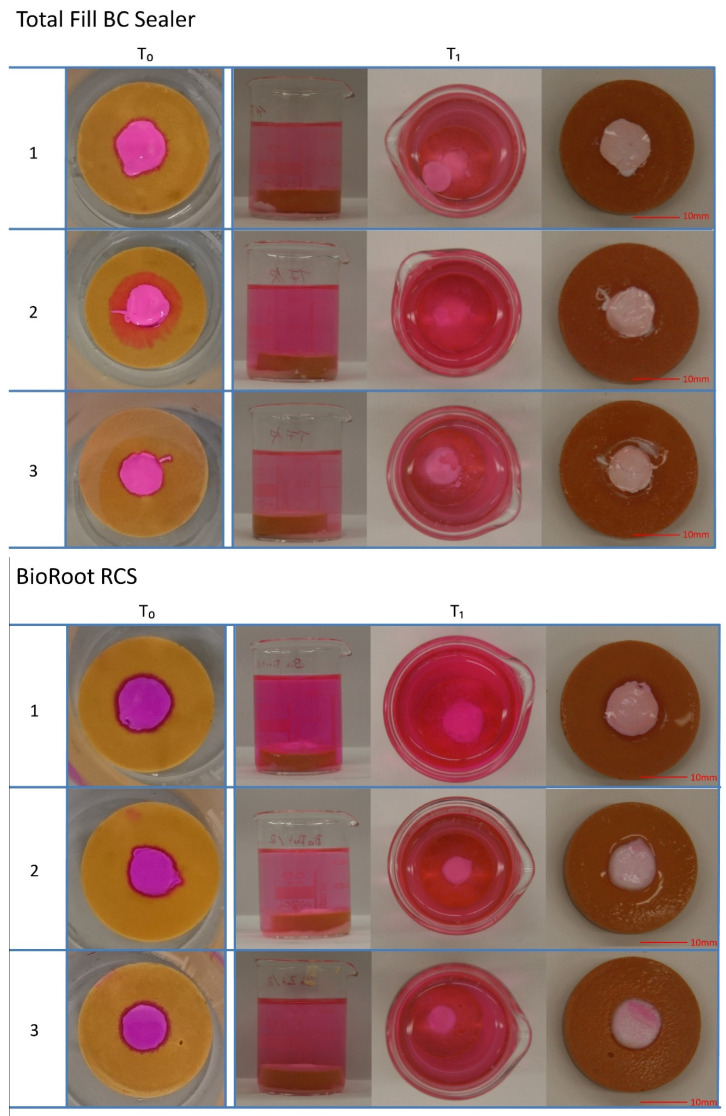
Photographs of the sealers AH Plus, Total Fill BC Sealer, and BioRoot RCS (*n* = 3 per sealer) at T_0_ (sealers mixed with rhodamine B) and T_1_ (sealer after contact with PBS for 7 days). AH Plus was not stained red by rhodamine B dye under dry conditions, while Total Fill BC Sealer and BioRoot RCS presented in a pink color directly after mixing. After the exposure of AH Plus to PBS, the surface turned red. Total Fill BC Sealer and BioRoot RCS lost their coloration after contact with PBS, and the buffer solution turned to a pink color.

The photographs of the sealers at T_0_ and T_1_ are presented in Figure 1. At T_0_, red staining of AH Plus with rhodamine B was not possible, as water is needed in order for rhodamine B to present its red color. Only after contact with PBS did the surface of AH Plus change to a red color. No coloration of the buffer solution was observed at T_1_. For both Total Fill BC Sealer and BioRoot RCS, a pinkish coloration of the sealers was observed at T_0_. After incubation in PBS for 7 days, the sealers Total Fill BC Sealer and BioRoot RCS nearly totally lost their coloration, while the buffer solutions were dyed red by the rhodamine B.

### 3.2. Experimental Setup 2

**Figure 2:**
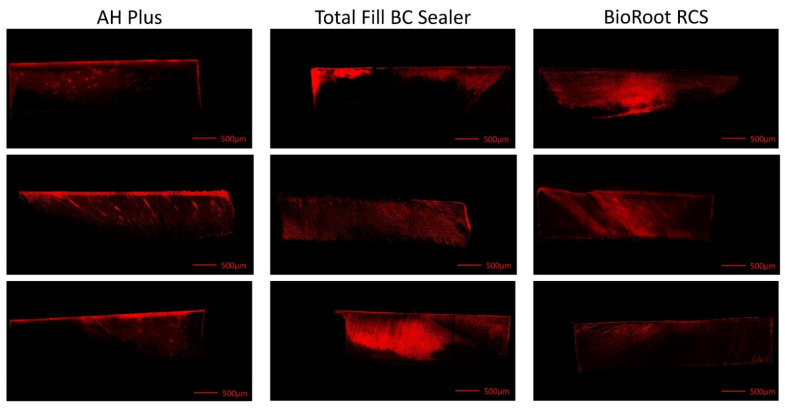
Confocal laser scanning microscope (CLSM) images of root specimens showing passive penetration of leached rhodamine B dye from AH Plus, Total Fill BC Sealer, and BioRoot RCS (*n* = 3 per sealer) into dentinal tubules. The sealers mixed with rhodamine B were placed on the top surface. Rhodamine B leached into the dentinal tubules and stained the dentinal tubules red.

The confocal images of the dentin specimens revealed penetration of rhodamine B dye along the dentinal tubules in all specimens, irrespective of the sealer used (Figure 2).

### 3.3. Experimental Setup 3

**Figure 3:**
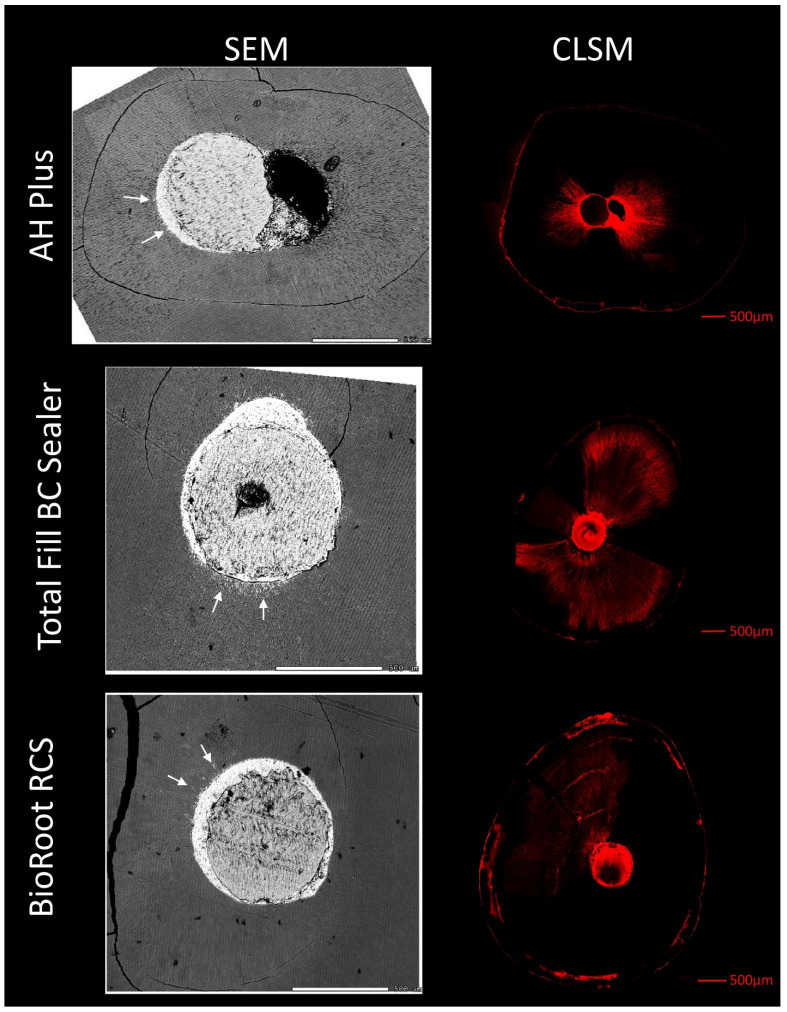
Corresponding scanning electron microscope (SEM) and confocal laser scanning microscope (CLSM) images of root slices filed with gutta-percha and AH Plus, Total Fill BC Sealer, and BioRoot RCS, as indicated. White arrows indicate the sealer penetration depth found in SEM images. Rhodamine B dye reached the root surface of all specimens in the CLSM images. No correlation between sealer penetration in SEM and rhodamine B penetration in CLSM could be detected. In SEM images, sealer presents as white, and can be detected via EDX analysis. Gutta-percha, which can also be detected via EDX analysis, presents as gray. Sealer penetration occurred only in areas with a high number of dentinal tubules.

Penetration of rhodamine B dye along the dentinal tubules was obvious in all specimens, irrespective of the sealer used (Figure 3). The penetration depth of rhodamine B dye was compared to sealer penetration at corresponding sites using SEM. Sealer penetration into the dentinal tubules in SEM images was far less than predicted by confocal laser imaging. Rhodamine B reached the root surface in all specimens in CLSM at some point, while sealer was only detected in a range of less than 100 µm from the root canal by SEM. No correlation between the two methods was observed.

No correlation between the two investigation methods concerning the dentinal tubule penetration depth of the sealers was observed.

## 4. Discussion

In the present study, rhodamine B dye leached from sealers even after the materials were fully set. Leaching was impressive, especially when calcium-silicate-based sealers were used. Rhodamine B leached from all sealer types investigated, in sufficient quantities to passively infiltrate the dentinal tubules. Therefore, the dentinal tubule penetration depth was overestimated when fluorescent dyes were used for CLSM investigation, as no such sealer penetration was detectable during SEM investigation. Thus, the null hypothesis was rejected.

To assess the penetration of the sealers into dentinal tubules, different techniques were described. Light microscopy was first used to visualize the sealer [16,17], but only the surface of the root slice could be subjected to investigation. Light microscopy was used because of caveats against the specimen preparation necessary for SEM evaluation [17]. For SEM evaluation, specimens had to be exsiccated in alcoholic solutions or high vacuum before examination. It was feared that this process might cause artifacts. Because of the concerns raised about SEM evaluation, no special specimen preparation techniques were performed in the present study. The specimens were desiccated at room temperature in a closed container for 3 months. In addition, no surface preparations—such as polishing—were performed, so as to avoid artifacts. In other studies, specimen surfaces had been polished [17], irrespective of the fact that this could also have pressed loose material into open tubules, indicating higher sealer penetration.

Due to concerns that specimen preparation for SEM evaluation or stereomicroscopy could remove sealer from the dentinal tubules at the specimen’s surface, CLSM was introduced to visualize the sealer [18]. It was argued that CLSM allows the evaluation of the optical section underneath the specimen’s surface, while no surface preparations that could cause artifacts, such as polishing or smear layer removal, are necessary.

A wide range of studies using the above-described technique has been published [3,4,5,6,17,19]. Though the variety of results showed a broad range, the penetration of sealers was profoundly deep in most studies. Maximum penetration of Total Fill BC Sealer of almost 2 mm deep into the root canal dentin was reported [6]. Mean penetration depths of AH Plus could be found in a range from 12 [5] to 984 µm [6]. The variety of results for calcium-silicate-based sealers was equally implausible [4,19,20]. Though different obturation techniques, chelating agents, and other comparable variables were under investigation in the studies, the different values reported could indicate a relevant error-proneness of the methodology. Irrespective of the anatomical variations in the specimens, such deep penetration of viscous material in small tubules presents a hydrodynamic miracle. In conclusion, the results of CLSM investigation show a variety that raises doubts about the reliability of the methodology. For CLSM investigation, materials must be labelled with a fluorescent dye. In the first study establishing the use of rhodamine B dye for sealer penetration investigation, it was stated that sealer penetration with and without rhodamine B staining was found to be similar in preliminary investigations [18]. Unfortunately, neither the methodology of the preliminary testing nor the exact results were included. Referring to the aforementioned study from 2007 [18], all further investigations published in recent years did not further verify this particular method. In another early study from 2007 on sealer penetration using the CLSM/rhodamine B technique, it was claimed that leaching of rhodamine B into the dentin could not occur [21]. In their argumentation, the authors referred to two studies that used rhodamine to label dentin bonding agents [22,23]. No verification for the use of rhodamine B to label resin-based sealers was reported with sufficient precision [17,20]. Moreover, a verification of the method for other sealer types is missing from the literature.

According to the systematic investigation in the present study, the unverified labelling of different sealer types with rhodamine B or other fluorescent dyes might be an even more intense confounding factor that could occur during SEM assessment. Not only did rhodamine leach from the materials in a sufficient amount (setup 1) and penetrate the tubules passively (setup 2), moreover, no correlation was observed between the penetration depth of the sealer during SEM evaluation and the penetration depth of rhodamine B during CLSM (setup 3). In addition to the flaws in the detection of sealer penetration by CLSM, the concept of sealers penetrating deeply into dentinal tubules should be re-evaluated. In studies so far, using the calcium-affine marker Fluo-3 to investigate the penetration depth of calcium-silicate-based sealers, it was shown that sealer penetration is impressively inferior compared to values that can be expected from studies using rhodamine B [10,11,12], which also corroborates the finding of the present study. As no changes in the radiopacity of the root dentin were observed after root canal filling with warm vertical obturation, it is unlikely that a considerable amount of the very radiopaque sealer was pressed into tubules. Moreover, no changes in the radiopacity of root canal dentin can be observed in a three-dimensional investigation of root canal fillings in µ-CT investigation [24]. To press a relatively viscous paste like a sealer into a dentinal tubule with a diameter of 1–3 µm [25], a high pressure must be applied. It is unlikely that these high pressures can be achieved during obturation. Additionally, due to the short period of setting time, the viscosity, and the particle size of the sealer materials, the capillary force effect seems unlikely to allow the sealer to penetrate through the dentin. In this light, the missing differences between dental tubule penetration after different obturation techniques [16] might also serve as an argument for the impossibility of precise and long-term staining of sealer materials.

Therefore, the whole concept of sealer penetration must be reconsidered. One of the next steps would be to repeat the experiments conducted in the present study using the maker Fluo-3 to prove that fluorescent dyes are not suitable for assessing sealer penetration into dentinal tubules. Further investigations into sealer penetration and methodological approaches by directly detecting the sealer in the tubules underneath a specimen’s surface are necessary. Furthermore, it seems likely that dentinal tubule penetration does not relevantly contribute to the sealing of the root canal after root canal obturation. Therefore, tubule penetration should be considered in combination with other factors, such as sealing ability and bond strength.

## 5. Conclusions

Staining of sealers using a fluorescent dye, such as rhodamine B, is an inadequate method for the evaluation of the sealers’ penetration depth into dentinal tubules. Rhodamine B diffuses passively into the dentinal tubules, because the staining of sealers does not include permanent connection of the dye to any of the epoxy-resin- or calcium-silicate-based sealers we investigated. When using this method, sealer penetration depth into dentinal tubules is considerably overestimated.

## Data Availability

The data presented in this study are available on request from the corresponding author after obtaining permission of authorized person.
